# Severe Case of Chronic Pelvic Pain Syndrome: Recovery after Injection of Procaine into the Vesicoprostatic Plexus—Case Report and Discussion of Pathophysiology and Mechanisms of Action

**DOI:** 10.1155/2018/9137215

**Published:** 2018-06-26

**Authors:** R. M. Kronenberg, S. M. Ludin, L. Fischer

**Affiliations:** Department of Neural Therapy, IKOM, University of Bern, 3010 Bern, Switzerland

## Abstract

We describe a patient with a 35-year history of a severe chronic pelvic pain syndrome (CPPS) that failed to adequately respond to various drug therapies and other treatments by different specialists. In addition to the ongoing chronic pain, he suffered from week-long episodes of increased pain with no discernible trigger. At the first consultation with us the patient was in a particularly severe pain phase. He was taking four different analgesically effective drugs. In terms of therapeutic local anesthesia (neural therapy), we performed suprapubic injection of procaine 1% with infiltration of the vesicoprostatic plexus. Just a few minutes later, the pain decreased significantly. To maintain and further increase the effect, we performed the injection six more times. The patient gradually reduced and stopped all drugs and remained free of pain and discomfort ever since. This is the first report of a successful therapeutic infiltration of the vesicoprostatic plexus using a local anesthetic (LA) in a patient with CPPS that has been refractory to different treatments for many years. A possible explanation may be that the positive feedback loops maintaining pain and neurogenic inflammation are disrupted by LA infiltration. This can lead to a new organisation (self-organisation) of the pain-processing systems.

## 1. Introduction

Chronic pelvic pain syndrome (CPPS)—also called chronic (abacterial) prostatitis (CP) in men—is a common clinical syndrome characterized by pain and functional urogenital disorders. The National Institute of Health (NIH) assigns CPPS to Category III prostatitis ((I) acute bacterial prostatitis, (II) chronic bacterial prostatitis, (III) chronic prostatitis/CPPS, and (IV) asymptomatic inflammatory prostatitis).

*Epidemiology*. The prevalence of CP/CPPS is 2–10% and highest in the fifth decade of life [1, 2].

*Symptomatology*. Typically, pain occurs deeply in the abdomen, the perineum, penis, and testes. It may cause symptoms such as dysuria, sensation of residual urine, permanent urgency, pollakisuria, nocturia, bladder obstruction with emptying disorders, and pathological stool urgency or foreign body sensation in the anus.

*Etiology.* The etiology of CPPS is unknown. There is no correlation of disorders with histological signs of inflammation of the prostate [1, 3].

*Diagnosis and Differential Diagnosis*. CPPS is an exclusion diagnosis without any internationally standardized diagnostic procedure. To be excluded there are, among others, chronic bacterial prostatitis, urethritis, urogenital malignancy, stricture, neurological disorders with impaired bladder function, and psychological factors.

*Treatment Options*. There is no international consensus on therapeutic strategy. Most patients are empirically given antibiotics on suspicion of bacterial prostatitis, often accompanied by alpha blockers. Further symptom-reducing drugs and measures are applied, as well as, in certain cases, physical therapy and psychological care [1]. In terms of efficacy, none of these therapies offer any significant benefit over placebo, and none of them can be recommended as monotherapy [4].

*Anatomy of the Autonomic Nervous System in the Lesser Pelvis of the Male [5].* The innervation of ureters, urinary bladder, seminal vesicle, and prostate occurs mainly via the autonomic nervous system. Its sympathetic and parasympathetic fibers intermingle in the inferior hypogastric plexus. The fibers connecting to the prostate and urinary bladder form near the organs the closely associated plexus vesicalis and prostaticus (“vesicoprostatic plexus”). In addition, nociceptive sympathetic afferents run parallel to the axons of visceral efferents [6].

## 2. Case Report

We report on a 55-year-old man who was diagnosed with CPPS by urology specialists from the university hospital and referred to us for pain treatment with local anesthetics (neural therapy).

### 2.1. History and Findings

At his first consultation with us, the patient reported pain and other ailments that began 35 years prior, after a party in a damp basement, without vanishing ever since. In the same night, pollakisuria and dysuria occurred, and the patient noted a permanently painful foreign body sensation in the areas of the prostate and anus, as well as perineally. Furthermore, he complained of a burning sensation in the urethra, a slightly reduced urinary stream, and nocturia of varying frequency. In addition to the ongoing chronic pain, the patient suffered from week- to month-long episodes of increased pain with no discernible trigger. Overall, the pain and other symptoms progressed over time.

Over the years, various specialist urological examinations were carried out and several attempts at treatment with various empirical antibiotic therapies and analgesics were made. Also, nerve stimulation therapy was applied, and a probatory surgical removal of both seminal vesicles and an extension surgery on the anus were performed. None of these measures resulted in any improvement in pain or other symptoms. The patient was then referred to us by the urologists for a probatory pain treatment with LA.

At the first consultation with us the patient was in a particularly severe pain phase. He complained of permanent pain and discomfort perineally and in the areas of the prostate, anus, and urethra, associated with pollakisuria, dysuria, and nocturia (more than ten times per night). Due to this the quality of life was impaired to a large degree. The patient was desperate and did not believe that he could be helped anymore.

For nine years he was taking an analgesically effective antiepileptic drug, Gabapentin, as well as the nonsteroidal anti-inflammatory drug Diclofenac, the opioid Oxycodone, and the pain-modifying tricyclic antidepressant, Amitriptyline.

The National Institutes of Health Chronic Prostatitis Symptom Index (NIH-CPSI) resulted in 39 points (pain: 18; urinary symptoms: 10; quality of life impact: 11). In rectal palpation, the patient felt pain in the lesser pelvis while the prostate was inconspicuous, as was the case in the recently performed sonography, in which 50 ml of residual urine had been detected. The PSA value was found to be 0.4 ng/ml.

### 2.2. Treatment and Further Course

Our treatment consisted of suprapubic injection of 5 ml each of 1% procaine on the right and the left with infiltration of the vesicoprostatic plexus (in line with neural therapy). In this injection, the puncture site is directly behind the pectineal line (pecten ossis pubis), 5 cm laterally to the center of the symphysis. The puncture direction is 45° both medially and caudally. The needle point needs always to remain extraperitoneal in the paravesical connective tissue, in which the vegetative nerve fibers are located. The penetration depth in the described patient was 7 cm, with the needle gauge being 23.5 (0.6 mm).

Just a few minutes after the first injection, the pain decreased significantly and persistently to a level the patient had not experienced in years (in his own terms: 90% improvement of all symptoms). In the following days, the patient experienced a further and lasting significant reduction in pain and other symptoms. At the next consultation after two weeks NIH-CPSI resulted in 11 points (pain: 3; urinary symptoms: 4; quality of life impact: 4). To maintain and further increase the effect, we performed the suprapubic injection of procaine a total of six more times, initially once a month, and bimonthly later. With each injection, the pains continued to decrease, up to freedom from pain and discomfort. Relapses occurred less frequently, were significantly lower in intensity and the duration was significantly shorter with an average of three days. Even the symptom-free intervals were getting longer. The patient reduced Diclofenac and Oxycodone on his own initiative and completely discontinued both drugs after the fifth consultation. After seven treatments, he was also able to stop Gabapentin and Amitriptyline and remained free of discomfort (NIH-CPSI: 0 points), which also had a positive effect on his mental and social integrity.

### 2.3. Adverse Effects

No adverse effects were observed.

## 3. Discussion

### 3.1. Pathophysiological Considerations

To our knowledge this is the first description of a successful treatment of refractory CPPS by infiltration of the vesicoprostatic plexus vesicoprostaticus with a LA.

In the emergence and maintenance of chronic pain and inflammation the sympathetic nervous system plays an important role. Various mechanisms are involved which sometimes amplify each other through positive feedback, resulting in functional and structural neuronal changes. The pathomechanisms described below are thought to be involved in CPPS, too.

#### 3.1.1. Reflex Response to Nociceptive Stimuli

Visceral and somatic nociceptive afferents converge in the spinal cord at the same multireceptive posterior horn neurons (wide dynamic range neurons/WDR neurons) [7, 8]. From there, the following circuitry is divergent: (1) via the side horn to vegetative nuclei with activation of sympathetic efferents innervating the visceral, cutaneous, and muscular system; (2) via the anterior horn to the skeletal musculature; (3) to the brain [9–11].

Nociceptive processes trigger a reflex response that is mediated predominantly by the sympathetic system and occurs via cutivisceral, viscerocutaneous, viscero-somatic-motor and reflex tracts [8, 12, 13]. In the corresponding projection zones, this can lead to pain, increased muscle tone, and dysregulation of the associated internal organ, as well as changes in circulation, increased skin turgor, and hyperalgesia of certain skin areas (peripheral sensitization). This further increases the sympathetic activity (positive feedback).

#### 3.1.2. Sympathetic-Afferent Coupling and Sympathetic Sprouting

The processes described below lead to further amplification of the above-mentioned positive feedback. An important factor in the development of these iterative loops is known as “sympathetic-afferent coupling” [14–18]. In pathological conditions, sensory coupling may occur between peripheral sympathetic efferent nerves and afferent nociceptive neurons by producing a kind of short circuit. Nociceptive afferents express adrenergic receptors and can thus become susceptible to norepinephrine [14] as a result the efferent sympathetic system gets connected with the afferent nociceptive system: now, enhanced sympathetic activity causes excitation of nociceptive afferents, thus generating the perception of pain.

The process known as sympathetic sprouting, too, leads to analogous positive feedback, in which sympathetic fibers form basket-like structures in the dorsal root ganglia (DRG) of the nociceptive afferents under inflammatory conditions [19]. In terms of positive feedback (iteration), additional minor stimuli (peripheral or central) may be sufficient in this situation to evoke severe pain [20].

#### 3.1.3. Synaptic Long-Term Potentiation (LTP)

Neuronal activity which is increased in the context of nociceptive processes can lead to* synaptic long-term potentiation* (LTP) at the level of the spinal cord [21] and in sympathetic ganglia [22] in the sense of a sensitization process. The synaptic transmission is altered, so that a consistent presynaptic stimulation can lead to a potentiation of the postsynaptic response. Such neuroplastic changes (“pain memory”) may cause pain to continue to be generated even after the cause of pain has disappeared. In fact, the patient reported that the pain increases occurred out of the blue, i.e., without somatic or mental triggers.

#### 3.1.4. Inhibitory Mechanisms

Gate Control Theory deals with the input control of the afferences in the posterior horn [23–25]. The activity of thick A*β* fibers inhibits via inhibitory interneurons the transition between the peripheral nociceptive afferents and the posterior horn neurons. The collateral, thinner A*δ* and C fibers on the other hand counteract this presynaptic inhibition, so that nociceptive stimuli are passed on unhindered (“the gate opens”). Descending pathways controlled by the cortex and brainstem normally exert presynaptic inhibition on the posterior horn neurons (“the gate closes”).

The common feature of inflammatory pain states is decreased descending inhibition [26]. Negative emotions, too, can counteract descending inhibition and thus result in increased transmission of nociceptive signals to the brain [27].

#### 3.1.5. Neuroimmunological Interaction

In addition to pain, inflammation is part of CPPS. The sympathetic nervous system influences immunological processes. Proinflammatory cytokines, under certain conditions, can induce the expression of *α*1A adrenoreceptors on immune cells, through which norepinephrine induces increased production of interleukin- (IL-) 6, IL-1*β*, tumor necrosis factor (TNF) *α*, and IL-8 [28, 29]. This in turn leads to an increased accumulation of neutrophilic granulocytes and triggers a humoral immune response [30]. In addition to norepinephrine, postganglionic sympathetic axons also secrete neuropeptides such as substance P [9] and various prostaglandins. Nociceptive C fibers also secrete peripherally neuropeptides such as substance P, neurokinin A, and calcitonin gene-related peptide (CGRP) [9, 26, 31]. These neuropeptides induce vasodilatation and increase vascular permeability with plasma extravasation [31–33], regulate smooth muscle cell tone, and have a direct proinflammatory effect (neurogenic inflammation) [26, 34]. In turn, IL-1*β* increases the synthesis of substance P [35] in sympathetic ganglia and stimulates its secretion from afferent nerve endings [36]. Lymphocytes and macrophages on their part produce and secrete substance P [37, 38], leading to further positive feedback loops of neurogenic inflammation.

### 3.2. Mechanisms of Action of the Therapeutic Injection of Local Anesthetics

The well-directed injection of local anesthetics (LA) (neural therapy) can be used to directly interfere with the described pathomechanisms at various levels [12].

Repeated use may allow autoregulation of the pain-processing systems due to the short-term interruption of neuronal reflex arcs pathologically trapped in positive feedback [20, 39]. Clinical observations suggest that this may also resolve structural* sympathetic-afferent coupling* [27]. It has been shown that LA, including procaine, reduce* sympathetic sprouting* in spinal ganglia with increased spontaneous activity [40, 41] and indirectly prevent the induction of* synaptic long-term potentiation* by inhibiting extracellular signal-regulated protein kinase [42]. The repeated LA-induced blockage of sensitized nociceptive afferent neurons also enables the modulation of plastic changes in the neuronal centers (“*pain memory*”) [10].

In the case of* pain-inhibiting systems (gate control)*, the goal must be to close the gate. This can be achieved with injection treatment by means of two mechanisms: (1) the pinprick activating the thick fibers and (2) the LA inhibiting the thin fibers [12]. Furthermore lidocaine and procaine at low concentration enhance the function of glycine receptors, of the predominant inhibitory receptors in the spinal cord, and of GABA_A_ receptors mediating presynaptic inhibition in the spinal cord [43].

LA also have anti-inflammatory properties and can regulate the sympathetically mediated* neurogenic inflammation* described above [12, 44]. Their effect unfolds on different levels of the inflammatory cascade and they influence the synthesis and release of different inflammatory mediators [44].

Finally, LA are also antibacterial, antiviral, and antifungal [44]. However, this comes into effect in CPPS only as an exception, as there is usually no infection involved.

#### 3.2.1. Advantages of Procaine

We use procaine for injection treatments because it offers some advantages over other LA. As a result, no long-term side effects have been reported in over 100 years [12, 45]. Newer, long-acting LA have a less favorable side-effect profile [45]. In addition, the effects of injection treatment do not depend on the pharmacological duration of action of LA. On the contrary, in order to enable an early autoregulation of the organism, it is important to interrupt the positive feedback in pain and inflammation as briefly as possible [12, 45]. In addition, in contrast to amide-structured LA, ester-structured procaine promotes microcirculation at the site of injection not just by the sympatholytic effect, but also by pharmacological action.

Due to the low diffusion capacity of procaine, the effect is locally limited and therefore easily controllable [12]. 95% of procaine is locally degraded by pseudocholinesterase, so that the metabolism is hardly burdened [45].

## 4. Conclusion

Our findings and considerations suggest that in the pathophysiology of CPPS numerous positive feedback loops (“circuli vitiosi” maintained by the sympathetic nervous system) play an important role in pain and neurogenic inflammatory processes. Positive feedback loops can be broken by means of injection treatments with procaine (neural therapy), which gives the pain-processing system the opportunity to reorganize. The described pathomechanisms and the mechanisms of action of local anesthetic injection treatment show a striking congruence, so that the latter appears to be the logical treatment for CPPS. It has to be confirmed by clinical trials, if the effectiveness of the presented new treatment option can be generalized.

## Abbreviations

CGRP: Calcitonin gene-related peptide
CP: Chronic prostatitis
CPPS: Chronic pelvic pain syndrome
DRG: Dorsal root ganglia
IL: Interleukin
LA: Local anesthetics
LTP: Long-term potentiation
TNF: Tumor necrosis factor.

## References

[1] Pontari M. (2017). *Chronic prostatitis/chronic pelvic pain syndrome*.

[2] Krieger J. N., Riley D. E., Cheah P. Y., Liong M. L., Yuen K. H. (2003). Epidemiology of prostatitis: New evidence for a world-wide problem. *World Journal of Urology*.

[3] Nickel J. C., Roehrborn C. G., O'Leary M. P., Bostwick D. G., Somerville M. C., Rittmaster R. S. (2007). Examination of the Relationship Between Symptoms of Prostatitis and Histological Inflammation: Baseline Data From the REDUCE Chemoprevention Trial. *The Journal of Urology*.

[4] Anothaisintawee T., Attia J., Nickel J. C. (2011). Management of chronic prostatitis/chronic pelvic pain syndrome: A systematic review and network meta-analysis. *Journal of the American Medical Association*.

[5] Schünke M., Schulte E., Schumacher U., Voll M., Wesker K. (2006). *Prometheus LernAtlas der Anatomie. 2. Auflage*.

[6] Jänig W., Vaitl D., Schandry R. (1995). Neuroanatomy and functions, organ regulations and sensations. *From the heart to the brain*.

[7] Zieglgänsberger W., Weinschenk S. (2010). Neuronale Plastizität, Schmerzgedächtnis und chronischer Schmerz. *Handbuch Neuraltherapie – Diagnostik und Therapie mit Lokalanästhetika*.

[8] Fischer L. (2003). Pathophysiologie des Schmerzes und Neuraltherapie. *Praxis*.

[9] Jänig W. (2006). *The integrative action of the autonomic nervous system*.

[10] Jänig W., Baron R., Fischer L., Peuker E. T. (2011). Pathophysiologie des Schmerzes. *Lehrbuch Integrative Schmerztherapie*.

[11] Handwerker H. O. (1999). *Einführung in die Pathophysiologie des Schmerzes*.

[12] Fischer L. (2014). *Neuraltherapie – Neurophysiologie, Injektionstechnik und Therapievorschläge. 4. vollständig überarbeitete Auflage*.

[13] Brügger A. (1980). *Die Erkrankungen des Bewegungsapparates und seines Nervensystems*.

[14] Baron R., Raja S. N., Malmberg A. B., Chaplan S. R. (2002). Role of adrenergic transmitters and receptors in nerve and tissue injury related pain. *Mechanisms and Mediators of Neuropathic Pain*.

[15] Jänig W., Baron R. (2003). Complex regional pain syndrome: mystery explained?. *The Lancet Neurology*.

[16] Jänig W., Koltzenburg M. (1991). Plasticity of sympathetic reflex organization following cross-union of inappropriate nerves in the adult cat. *The Journal of Physiology*.

[17] Jänig W., Koltzenburg M., Jänig W., Schmidt R. F. (1992). Pathophysiological Mechanisms and Clinical Implications. *Reflex Sympathetic Dystrophia. Pathophysiological Mechanisms and Clinical Implications*.

[18] Jänig W., Levine J. D., Michaelis M. (1996). Interactions of sympathetic and primary afferent neurons following nerve injury and tissue trauma. *Progress in Brain Research*.

[19] Ramer M. S., Bisby M. A. (1997). Rapid sprouting of sympathetic axons in dorsal root ganglia of rats with a chronic constriction injury. *PAIN*.

[20] Fischer L., Baron R., Koppert W., Strumpf M., Willweber-Strumpf A. (2013). Neuraltherapie. *Praktische Schmerztherapie*.

[21] Sandkühler J. (2000). Learning and memory in pain pathways. *PAIN*.

[22] Alkadhi K. A., Alzoubi K. H., Aleisa A. M. (2005). Plasticity of synaptic transmission in autonomic ganglia. *Progress in Neurobiology*.

[23] Melzack R., Wall P. D. (1965). Pain mechanisms: a new theory. *Science*.

[24] Katz J., Rosenbloom B. N. (2015). The golden anniversary of Melzack and Wall's gate control theory of pain: Celebrating 50 years of pain research and management. *Pain Research & Management*.

[25] Braz J., Solorzano C., Wang X., Basbaum A. (2014). Transmitting Pain and Itch Messages: A Contemporary View of the Spinal Cord Circuits that Generate Gate Control. *Neuron*.

[26] Mense S. (2001). Pathophysiology of low back pain and the transition to the chronic state - Experimental data and new concepts. *Der Schmerz*.

[27] Fischer L., Weinschenk S. (2010). Schmerzgedächtnis und Neuraltherapie. *Handbuch Neuraltherapie – Diagnostik und Therapie mit Lokalanästhetika*.

[28] Heijnen C. J., Rouppe van der Voort C., Van de Pol M., Kavelaars A. (2002). Cytokines regulate *α*1-adrenergic receptor mRNA expression in human monocytic cells and endothelial cells. *Journal of Neuroimmunology*.

[29] Perez D. M., Papay R. S., Shi T. (2009). Alpha1-Adrenergic Receptor Stimulates Interleukin-6 Expression and Secretion through Both mRNA Stability and Transcriptional Regulation: Involvement of p38 Mitogen-Activated Protein Kinase and Nuclear Factor-KappaB. *Molecular Pharmacology*.

[30] Elenkov I. J., Wilder R. L., Chrousos G. P., Vizi E. S. (2000). The sympathetic nerve—an integrative interface between two supersystems: the brain and the immune system. *Pharmacological Reviews*.

[31] Lembeck F., Holzer P. (1979). Substance P as neurogenic mediator of antidromic vasodilation and neurogenic plasma extravasation. *Naunyn-Schmiedeberg's Archives of Pharmacology*.

[32] Brain S. D., Williams T. J., Tippins J. R., Morris H. R., MacIntyre I. (1985). Calcitonin gene-related peptide is a potent vasodilator. *Nature*.

[33] Buckley T. L., Brain S. D., Rampart M., Williams T. J. (1991). Time‐dependent synergistic interactions between the vasodilator neuropeptide, calcitonin gene‐related peptide (CGRP) and mediators of inflammation. *British Journal of Pharmacology*.

[34] Papathanasiou G., Weinschenk S., Weinschenk S. (2010). Periphere und spinale Modulation von Schmerz. *Handbuch Neuraltherapie Diagnostik und Therapie mit Lokalanästhetika*.

[35] Freidin M., Kessler J. A. (1991). Cytokine regulation of substance P expression in sympathetic neurons. *Proceedings of the National Acadamy of Sciences of the United States of America*.

[36] Inoue A., Ikoma K., Morioka N. (1999). Interleukin-1*β* induces substance P release from primary afferent neurons through the cyclooxygenase-2 system. *Journal of Neurochemistry*.

[37] De Giorgio R., Tazzari P. L., Barbara G., Stanghellini V., Corinaldesi R. (1998). Detection of substance P immunoreactivity in human peripheral leukocytes. *Journal of Neuroimmunology*.

[38] Lai J.-P., Douglas S. D., Ho W.-Z. (1998). Human lymphocytes express substance P and its receptor. *Journal of Neuroimmunology*.

[39] Jänig W., Fischer L., Peuker E. T. (2011). Rolle von motorischen Rückkopplungsmechanismen in der Erzeugung von Schmerzen. *Lehrbuch Integrative Schmerztherapie*.

[40] Takatori M., Kuroda Y., Hirose M. (2006). Local anesthetics suppress nerve growth factor-mediated neurite outgrowth by inhibition of tyrosine kinase activity of TrkA. *Anesthesia & Analgesia*.

[41] Zhang J.-M., Li H., Munir M. A. (2004). Decreasing sympathetic sprouting in pathologic sensory ganglia: a new mechanism for treating neuropathic pain using lidocaine. *PAIN*.

[42] Tan Z., Dohi S., Ohguchi K., Nakashima S., Nozawa Y. (1999). Local anesthetics inhibit muscarinic receptor-mediated activation of extracellular signal-regulated kinases in rat pheochromocytoma PC12 cells. *Anesthesiology*.

[43] Hara K., Sata T. (2007). The effects of the local anesthetics lidocaine and procaine on glycine and *γ*-aminobutyric acid receptors expressed in Xenopus oocytes. *Anesthesia & Analgesia*.

[44] Cassuto J., Sinclair R., Bonderovic M. (2006). Anti-inflammatory properties of local anesthetics and their present and potential clinical implications. *Acta Anaesthesiologica Scandinavica*.

[45] Gold-Szklarski K., Weinschenk S., Weinschenk S. (2010). Procain oder Lidocain? Die Verwendung von ester- oder amidstrukturierten Lokalanästhetika. *Handbuch Neuraltherapie – Diagnostik und Therapie mit Lokalanästhetika*.

